# Porosity Tunable Metal-Organic Framework (MOF)-Based Composites for Energy Storage Applications: Recent Progress

**DOI:** 10.3390/polym17020130

**Published:** 2025-01-08

**Authors:** Huddad Laeim, Vandana Molahalli, Pongthep Prajongthat, Apichart Pattanaporkratana, Govind Pathak, Busayamas Phettong, Natthawat Hongkarnjanakul, Nattaporn Chattham

**Affiliations:** 1 Department of Physics, Faculty of Science, Kasetsart University, Bangkok 10900, Thailand; huddad.lae@ku.th (H.L.); fsciacp@ku.ac.th (A.P.); 2 Department of Physics, B.M.S. College of Engineering, Bull Temple Road, Bengaluru 560019, India; vandanam254@gmail.com; 3 Centre for Nano-Materials and Displays, B.M.S. College of Engineering, Bull Temple Road, Basavanagudi, Bengaluru 560019, India; 4 Department of Materials Science, Faculty of Science, Kasetsart University, Bangkok 10900, Thailand; fscipop@ku.ac.th; 5 Geo-Information and Space Technology Development Agency (GISTDA), Sriracha 20230, Thailand; govindpathak001@gmail.com (G.P.); busayamas@gistda.or.th (B.P.); natthawat@gistda.or.th (N.H.)

**Keywords:** metal–organic framework, energy storage, supercapacitor and batteries, porous coordination polymers

## Abstract

To solve the energy crisis and environmental issues, it is essential to create effective and sustainable energy conversion and storage technologies. Traditional materials for energy conversion and storage however have several drawbacks, such as poor energy density and inadequate efficiency. The advantages of MOF-based materials, such as pristine MOFs, also known as porous coordination polymers, MOF composites, and their derivatives, over traditional materials, have been thoroughly investigated. These advantages stem from their high specific surface area, highly adjustable structure, and multifunctional nature. MOFs are promising porous materials for energy storage and conversion technologies, according to research on their many applications. Moreover, MOFs have served as sacrificial materials for the synthesis of different nanostructures for energy applications and as support substrates for metals, metal oxides, semiconductors, and complexes. One of the most intriguing characteristics of MOFs is their porosity, which permits space on the micro- and meso-scales, revealing and limiting their functions. The main goals of MOF research are to create high-porosity MOFs and develop more efficient activation techniques to preserve and access their pore space. This paper examines the porosity tunable mixed and hybrid MOF, pore architecture, physical and chemical properties of tunable MOF, pore conditions, market size of MOF, and the latest development of MOFs as precursors for the synthesis of different nanostructures and their potential uses.

## 1. Introduction

The energy crisis has recently brought to light a major social issue that impedes progress and ultimately threatens human life [1]. With the global economic boom, there has been a sharp increase in the demand for new and alternative energy sources worldwide. This demand is primarily driven by growing global concerns about environmental issues like climate change and global warming, which includes melting glaciers and rising sea levels, as well as the sustainability of fuel supplies. Thus, the creation of energy storage and conversion technologies that are safe, clean, renewable, and sustainable has gained popularity as a study area. The scientific community has therefore shown a great deal of interest in the advancement of the design of clean, safe, sustainable energy storage devices (such as batteries and supercapacitors) and conversion technologies (such as fuel cells) [2,3,4]. Because of their great energy efficiency and environmentally benign power systems, electrochemical energy storage devices have garnered a lot of attention in recent times. To actualize these innovative applications, it is necessary to use materials with optimal designs and relevant functionality. Because of their exceptional superiority over conventional materials for energy conversion and storage applications, metal–organic framework (MOF)-based materials, such as pristine MOFs, MOF composites, and MOF derivatives, have attracted a lot of attention among developing materials.

Using strong coordinate bonds, different metal nodes (such as ions or clusters) and organic ligands self-assemble to form pristine MOFs, sometimes referred to as porous coordination polymers. The advantages of a highly porous structure, structural variety, utility, and tailorability can thus be demonstrated by pure MOFs. Moreover, post-modification techniques can be used to modify pure MOFs in order to expand their range of uses. For example, MOFs can be used as functional support with other auxiliary elements to form MOF composites with standardized architectures for different uses. MOFs can also be used as stand-in precursors to create materials derived from MOFs, like metal compounds, porous carbon materials, and their composites. These materials have tunable active sites that perform better in electrochemical storage and conversion.

**Figure 1:**
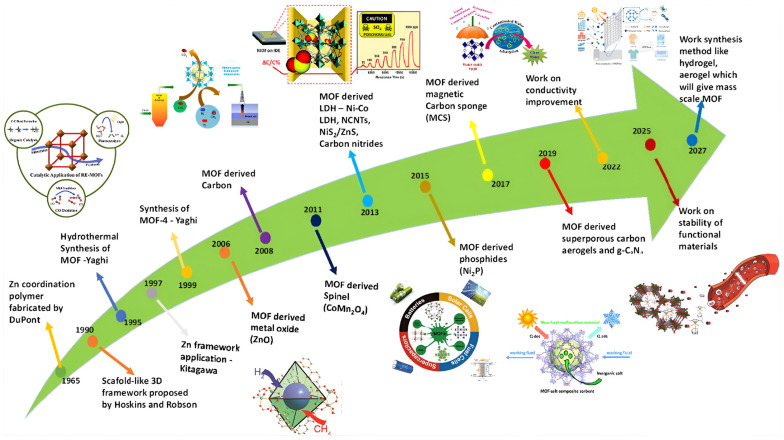
The development of metal–organic frameworks and their prospects for high-performance supercapacitors [5].

Metal–organic frameworks (MOFs) have emerged as an effective class of porous organic–inorganic ordered structures among a variety of cross-functional platforms. MOFs’ unique properties position them as pivotal building blocks for crafting cutting-edge energy materials. The recent developments and future research of MOF-based material for energy, starting from 1965 to 2027, are shown in Figure 1.

**Figure 2:**
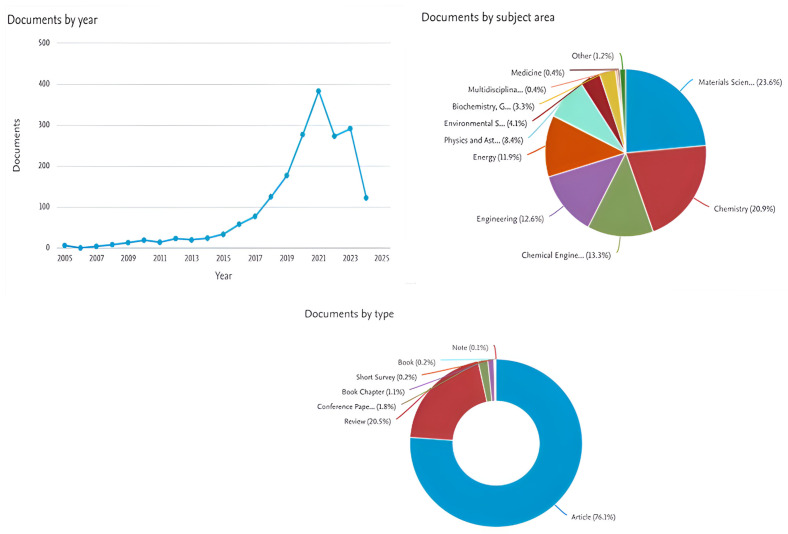
The Web of science data of MOF-based material for energy applications (typing keyword MOF, Energy) and number of documents published by type, subject area and the year.

Due to their abundant porosity, numerous redox sites, and expansive surface area, metal–organic frameworks (MOFs) have garnered significant attention and were extensively explored as effective electrode materials for supercapacitors (SCs). Nevertheless, two significant obstacles to the continued use of MOF-based materials in energy storage applications are their aggregated structures and low conductivity. Most MOFs are converted into functional materials to address low conductivity and weak electrochemical active sites. These materials require energy-intensive processes, such as high temperature annealing, which frequently results in reduced porosity and low surface area. Therefore, developing an effective and systematic method is essential for synthesizing MOFs as improved electrode materials to address these issues. The web of science data of recent developments of MOF-based materials for energy devices is shown in Figure 2. The number of documents published from 2005 to 2024, documents in various subject areas in percentage, and document type shows that more working articles were published compared to books and reviews. This shows that more and more research is occurring in this field.

There are still difficulties in using MOF-based materials, even though their remarkable qualities have drawn scientists to investigate them thoroughly, and their uses in the energy sector are widely documented. For instance, the low fuel storage utilization and low apparent quantum yield (AQY) of MOF-based materials still need to meet the requirements of real-world applications. In this review, we provide an overview of the recent advancements in the utilization of MOF-based materials across various energy-related sectors over the past several years.

The review paper that is currently available provides an overview of MOF materials that have micropores, mesopores, and macropores in various configurations with multi-range porosities. The pore sizes and habitats of MOF resources meant for battered actions can be altered hierarchically, which enhances the overall framework discernment. Therefore, increasing the porosity in a hierarchical manner will enable more effective substrate transmission within the pore structures.

The review focused on pristine MOFs, composites, and derivatives, as introduced first. Next, we discuss the synthesis techniques for MOF derivatives and their composition, architecture, and MOF nanoarchitectures, such as 0D nanoparticles, 1D nanorods, 2D nanosheets, and 3D hierarchical structures. Finally, we provide a thorough and up-to-date assessment of their use in a variety of energy technologies, such as gas storage, supercapacitors, rechargeable batteries, and electrochemical energy conversion. We provide the most recent developments in the use of MOF-based materials as well as a comparative analysis of them. The goal of this review is to compile the most recent and useful data so that scientists may better grasp the benefits and difficulties of using MOF-based materials in various energy situations, maximize their utility value, and expand their applications.

This review’s novelty examines the creative applications of metal–organic frameworks (MOFs) as essential parts of supercapacitor technology. Significant improvements are possible with the incorporation of MOFs into supercapacitors. The potential of MOF-based supercapacitors in next-generation technologies like electric vehicles or hybrid energy storage systems (such as supercapacitors) was examined in this research. Thus, this analysis concentrates on their cutting-edge performance, current advancements, and prospects for energy storage technology in the future.

## 2. Pristine MOFs, Composites, and Derivatives

Since Yaghi and Li introduced the idea of MOFs in 1995 [6]; thousands of MOFs were found and created throughout the last few decades. Metal ions/metal clusters and polydentated organic ligands combine to form MOFs, which are self-assembling coordination polymers. MOFs exhibit an adjustable crystal structure, open metal sites, adjustable pores, and a high specific surface area. Sensing, catalysis, ion exchange, adsorption/separation, gas storage, and energy storage are among the many applications for which they are employed [7]. The study of MOFs for energy conversion and storage is still in its infancy. Most of the research has focused on employing materials derived from MOFs in energy conversion and storage domains, including metal oxides, porous carbon, and carbon-based metal nanoparticles [8]. However, because of their low electronic conductivity, using pristine MOFs directly as electrode materials is quite difficult.

**Figure 3:**
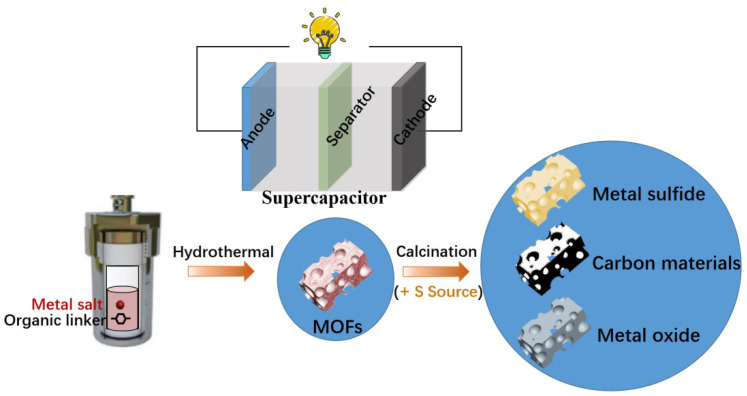
Utilization of metal–organic frameworks and their derivatives in supercapacitors [10].

The physical and chemical architectures of MOFs can be optimized by a variety of organic ligands and reaction circumstances with a planned porosity structure and different morphologies when using MOF materials as electrodes for energy storage [9]. Furthermore, MOFs have a lot of different ligands and metal ions or clusters, which provide a lot of redox sites for electrochemical processes. Consequently, the electrochemical performance of MOFs and the materials from which they are produced is greatly influenced by their pores, architectures, and crystal shapes [9]. While most MOFs have low conductivity, this can be changed by building conjugate systems and 3D network architectures. This allows for the direct use of well-designed, pure MOFs as materials for electrodes in energy conversion and storage devices, including SCs shown in Figure 3.

Using a one-step hydrothermal process, Zhang et al. [11] created spherical NiCo-Bimetallic electrode (Ni: Co ratio of 2:1) composed of ultrathin nanosheets. The fabricated NiCo- Bimetallic electrode, in 2 mol/L KOH electrolyte, exhibits a greater Csp (568 C g^−1^ at 1 A g^−1^) and much better cycle stability (75.5% retention over 3000 charge/discharge cycles) compared to pure Ni- Bimetallic (SC: 407 C g^−1^ at 1 A g^−1^, retention: ~35% after 3000 cycles). This is due to the beneficial combination of unique structures and mixed metallic components. With reduced graphene oxide as the negative electrode and NiCo-Bimetallic as the positive electrode, the assembled asymmetric supercapacitor achieves an impressive energy density of 42.24 Wh kg^−1^ at a power density of 800 W kg^−1^, demonstrating exceptional electrochemical stability while cycling (82.6% of the retention of initial capacitance over 6000 charge/discharge cycles).

**Figure 4:**
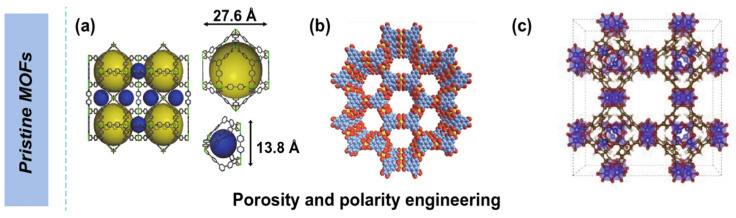
Development of MOF materials for upcoming generation rechargeable energy sources [14]. Figure (**a**) Structure of Ni –MOF, (**b**) Crystal structure of Ni_3_(HITP)_2_. (**c**) crystal structure of MOF-199.

Pristine MOFs offer advantages such as high polarity, rich pores with defined morphology, and compatibility with active sulfur, reducing volume variation and preventing polysulfide shuttling. They also provide protection for the Li metal anode. Tarascon et al. (2011) introduced mesoporous MIL-100 (Cr) as a pioneering sulfur host for Li–S batteries [12]. Although 48% sulfur incorporation into the high surface area MIL-100 (Cr) was achieved, weak binding between oxygenated MOF groups and polysulfides led to poor cycle stability in batteries. In 2014, Xiao et al. proposed a microporous and mesoporous Ni-MOF (Ni6(BTB)4(BP)3; where BTB = benzene-1,3,5-tribenzoate and BP = 4,4′ bipyridyl)) and Ni(II) acted as a Lewis acid and polysulfides as a Lewis base [13] (Figure 4a). The Ni-MOF effectively traps polysulfides through both physical and chemical interactions, enhancing the cycling stability of the sulfur cathode. This is evidenced by a remarkable capacity retention of 89% after 100 cycles at 0.1 °C. However, the insulating properties of MOFs lead to suboptimal sulfur utilization, and the framework may degrade over time, especially with prolonged cycling.

Large-surface MOFs with adjustable porosity should be good ionic sieve candidates to block the shuttling of polysulfide ions. To mitigate the issue of polysulfide shuttling, Fang and colleagues demonstrated the production of a conductive and microporous MOF, Ni3(HITP)2 (HITP = 2, 3, 6, 7, 10, 11-hexaiminotriphenylene), with polysulfide-capturing capability directly on the separator (Figure 4b) [15]. When employed in Li–S batteries with a high sulfur loading of 8.0 mg cm^−2^, the functional separator made of MOFs achieved a significant surface capacity of 7.24 mAh cm^−2^ after 200 cycles at 0.5 °C, retaining 86% of its capacity. Afterward, a straightforward wet-chemistry technique was created to produce thin MOF (Cu_2_(CuTCPP)) nanosheets [16]. The Li–S batteries employing a MOF-based separator exhibited remarkable durability, enduring 2000 cycles with a minimal capacity degradation rate of 0.015% per cycle. Recent work by Chen’s group revealed that MOF-199 effectively shields against Li dendrite formation and promotes uniform Li+ concentration through its porous structure (Figure 4c) [17]. Nevertheless, MOFs’ inherent mechanical brittleness prevented them from meeting the practical needs of stable and long-lasting Li–S batteries.

**Figure 5:**
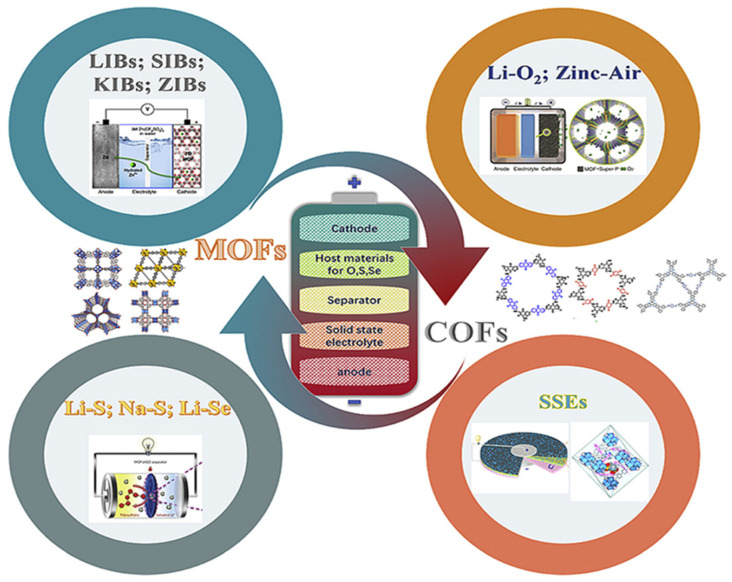
Perfect MOF and covalent organic framework (COF) components for cutting-edge batteries [19].

Types of crystalline porous organic–inorganic hybrid MOF compounds suggested by Zibin Liang et al. [7] have recently drawn more interest in the realm of energy conversion and storage. This article summarizes recent developments in MOFs and MOF composites for energy storage and conversion applications, such as Li-based batteries (Li-ion, Li–S, and Li–O_2_ batteries), water oxidation, supercapacitors, and photochemical and electrochemical fuel generation (hydrogen production and CO_2_ reduction). Typical development approaches for MOFs and composites made of them for particular energy storage and converting applications are discussed, such as the addition of active elements and the creation of clever morphologies. To potentially encourage further research of MOFs and composites made of them for improved storage of energy and conversion applications, a wide overview of current accomplishments is presented in Figure 5.

To enhance the electro/photochemical properties and adsorption efficacy of porous MOF materials for H_2_ generation, storage, and utilization, Paitandi et al. proposed design strategies [18]. Lastly, challenges and future pathways for developing relevant MOFs and MOF composites in the context of the H_2_ economy are addressed.

**Figure 6:**
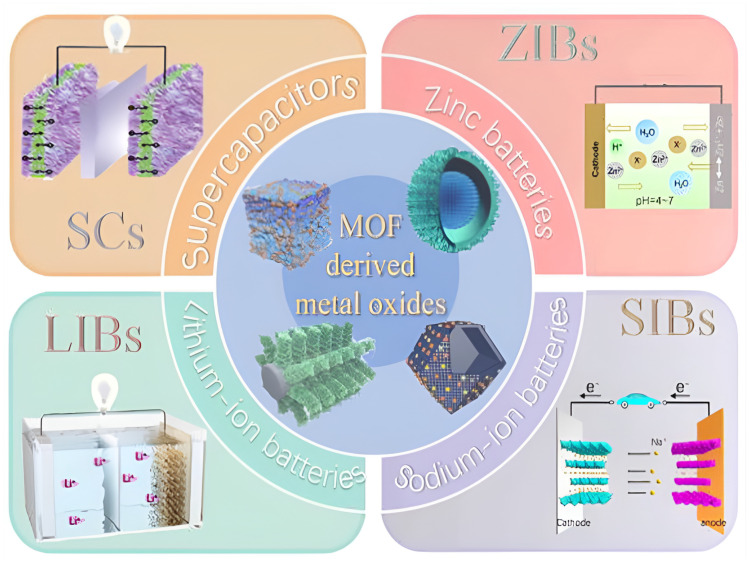
Metal oxide composites produced from MOFs and their potential uses in energy storage [19].

Chao Li et al. recently underscore pristine MOFs and covalent organic frameworks (COFs) within three crucial battery components: electrode materials (or host materials), solid-state electrolytes, and separators We offer insights into the benefits and challenges of this evolving field, delve into the underlying mechanisms, and propose design criteria for real-world battery applications shown in Figure 6.

## 3. Porosity Tunable Mixed Pristine and Hybrid MOF Materials

The most notable qualities of MOFs are presumably their exceptional porosity and surface area. By choosing the right linkers and metal nodes, the framework’s pore size and topology may be precisely adjusted. A library of linked organic linkers with varying lengths and functionality is used to create isoreticular MOFs, which are characterized as frameworks with the same structural topology [20]. It is possible to study structural and chemical parameters that influence electrochemical processes independently thanks to the remarkable control over pore size and chemical environment in isoreticular MOFs. Frameworks with a mix of micropores and mesopores, known as hierarchically porous MOFs, further produce pore space and tactical channels that might affect molecule diffusion. Oxygen reduction (ORR) in lithium-oxygen batteries is one catalytic mechanism that benefits from a higher surface area. This unique capacity to adjust surface area and porosity in traditional inorganic solids opens up new possibilities for electrochemical applications.

The International Union of Pure and Applied Chemistry (IUPAC) has recommended that porous materials, including zeolites, activated carbon, and metal oxide fillers (MOFs), be categorized into three groups based on the size of their pores: macroporous, mesoporous, and microporous [21].

Materials classified as macroporous have pores larger than 50 nm, mesoporous have pores between 2 and 50 nm, and microporous have pores narrower than 2 nm. Interpreting the isotherms of flexible MOFs is a challenging task due to their structural alteration throughout the adsorption process. As a result, it is imperative to continue developing cutting-edge methods for identifying, analyzing, and categorizing novel physisorption isotherms seen in flexible MOFs. Once MOFs have established permanent porosity, gathering physisorption isotherms has become a standard procedure for characterizing MOF materials. Surface area, pore volume, and pore size distribution can all be obtained by interpreting a physisorption isotherm, such as an argon or nitrogen adsorption isotherm. The Brunauer–Emmett–Teller (BET) method is frequently used to calculate the surface area of porous MOFs. It is based on a multilayer adsorption model.

In practice, it is advised to minimize the departure from these criteria when it is not possible to choose a region from experimental isotherms that satisfies all four BET consistency criteria. It is significant to note that many reported BET area values only meet the first two BET consistency requirements. However, we think the trend will likely be to apply all four BET consistency criteria, especially for highly porous MOFs with large surface areas. BET area, in spite of its drawbacks, offers a widely recognized comparison of MOF surface areas and serves as a useful indicator of MOF adsorbents. Another crucial factor in determining the porosity of MOFs is pore volume.

The chemistry and structure of the pores play a significant role in determining the characteristics and uses of MOFs. Significant advancements in the synthesis processes and characterization methods in the fields of material science, reticular chemistry, and organic synthesis chemistry have made it possible to tune MOF-based materials’ chemistry and pore sizes in a flexible manner, expanding the materials’ potential uses.

**Figure 7:**
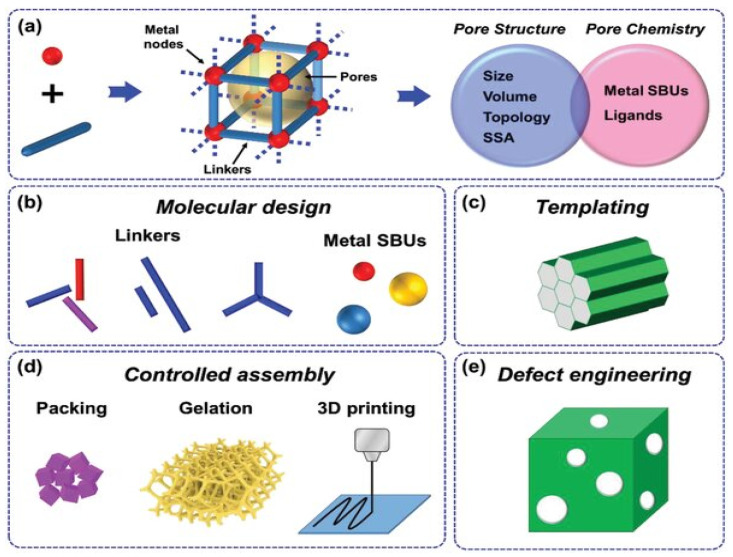
Methods for pore architecturing MOFs (**a**) Structural features and design directions of MOFs. (**b**–**e**) General strategies for manipulating pores, including (**b**) molecular design, (**c**) templating, (**d**) controlled assembly, and (**e**) defect engineering. [22].

Understanding coordination chemistry, reticular chemistry, and traditional organic chemistry are necessary for the synthesis of MOFs. The development of MOFs, as shown in Figure 7a, can be understood as the assembly of lines (i.e., linkers) and polyhydra (i.e., metal nodes) through coordinating interactions, resulting in the formation of long-range-ordered frameworks with particular topologies. Because of the reduced amounts of bonds and dispersive interactions between the voids, MOFs are thermodynamically unstable with regard to their dense phase. This is because the pores tend to collapse. However, in order to account for the stability of MOFs, metastability can be accomplished by increasing the energy barrier through the use of inert metal ions, strong metal–ligand interactions, and improved connectivity between linkers and nodes.

**Figure 8:**
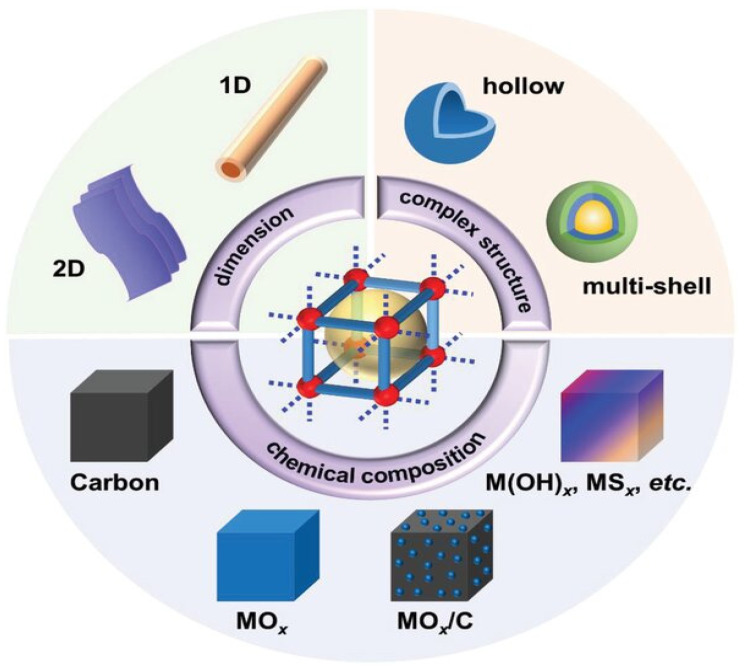
Strategies for pore architecturing materials generated from MOFs.

With the right processing, pristine MOFs can be converted into MOF derivatives. A range of porous materials, such as metal oxides (MOx), metal sulfides (MSx), metal selenides (MSex), metal phosphides (MPx), metal nitrides (MNx), their carbon-based composites, pure carbon, and metal hydroxides (M(OH)x), can be produced by choosing the appropriate MOF precursors and managing processing parameters. These materials typically have enhanced electrical conductivity. The pore architecturing techniques for these MOF derivatives can be broadly categorized into two categories: pore chemistry, which involves diverse chemical compositions, and pore structure design, which involves various pore volumes, specific surface areas (SSAs), dimensions, and complex architectures. These techniques are similar to those used for pristine MOFs, as shown schematically in Figure 8. Additionally, efforts were recently made to create different kinds of porous materials from MOFs for use in supercapacitors. Using metal double hydroxides (MDHs), which are produced by processing MOFs with potassium hydroxide, is a common example.

A variety of MOF-74 were generated by varying the ratio of nickel to cobalt, which is subsequently converted to the equivalent MDH (X-Ni-MDH, where X is the percentage of Ni in MOF-74). The optimized formula, 65Ni-MDH with 65% Ni and 35% Co, performs better than MDHs with all other compositions, with exceptional specific capacitance and rate performance (875 and 666 C g^−1^; that is, 1750–1332 F g^−1^ at 1–20 A g^−1^) and good long-term stability (90.1% retention after 5000 cycles) [23].

**Figure 9:**
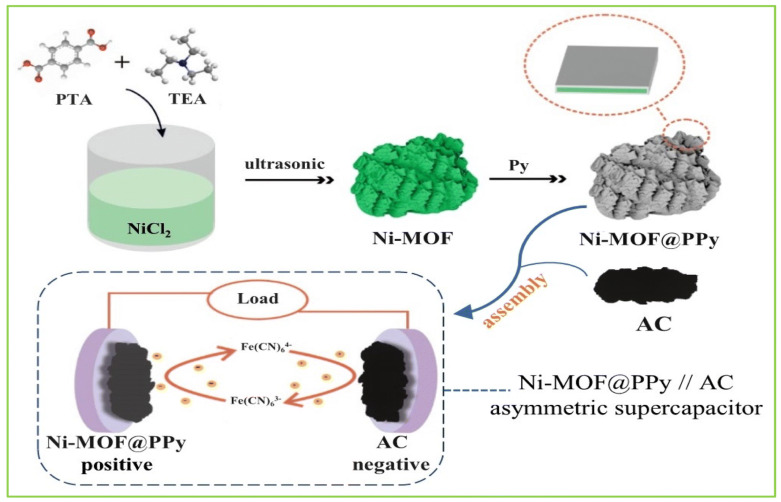
The production of Ni-MOF@PPy and its application in the construction of the asymmetric supercapacitor Ni-MOF@PPy/AC [24].

The electron transport properties of metal–organic frameworks (MOFs) still provide a challenge to supercapacitor electrodes. Thus, to enhance its electron transfer property, a Ni-based metal–organic framework (Ni-MOF) doped with poly-pyrrole (PPy) was converted into an electrode composite material, Ni-MOF@PPy, using a straightforward chemical oxidation process shown in Figure 9. Asymmetric supercapacitors in the KOH and K_4_Fe(CN)_6_ mixed electrolyte were assembled using Ni-MOF@PPy and active carbon (AC) as the positive and negative electrodes, respectively. This new Ni-MOF@PPy//AC ASC energy storage device has a 90.2% capacitance retention rate, 7001 W kg^−1^ power density, and 38.5 Wh kg^−1^ energy density.

**Figure 10:**
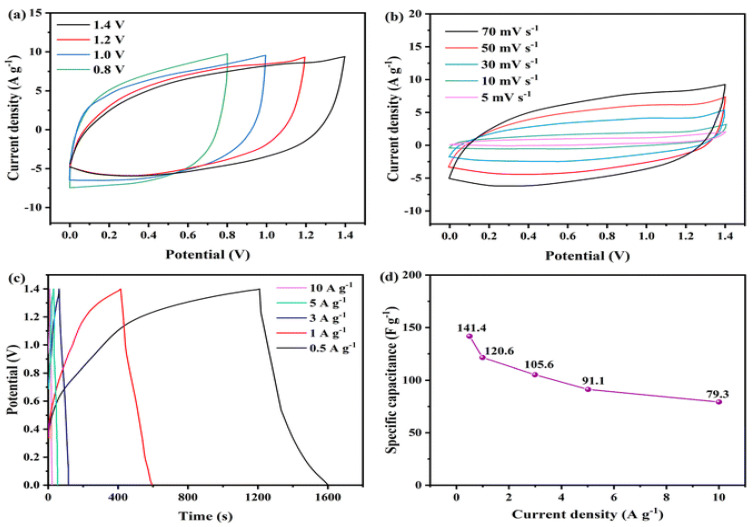
The electrochemical properties of Ni-MOF@PPy//AC ASC are demonstrated by the following: (**a**) specific capacitance curve at various current densities; (**b**) CV curves under varying scan rates; (**c**) GCD curves at various current densities; and (**d**) CV curves at various voltage windows [23].

Initially, Ni-MOF@PPy//AC ASC underwent CV testing at a scanning rate of 70 mV s^−1^ across different potential ranges (0–0.8, 0–1.0, 0–1.2, and 0–1.4 V) Figure 10a. The CV curve of the supercapacitor exhibited double-layer characteristics, and upon extending the voltage window to 1.4 V, Faraday redox behavior was observed, without noticeable polarization. These results indicate a voltage window of 1.4 V for the system. Figure 10b illustrates CV curves at varying scanning rates, demonstrating minimal distortion even at rates up to 70 mV s^−1^, suggesting highly reversible electrochemical processes with low resistance [25]. During the GCD test, ASC devices reached a voltage of 1.4 V Figure 10c,d. The specific capacitance of ASC devices was found to be 141.4, 120.6, 105.6, 91.1, and 79.3 F g^−1^ at current densities of 0.5, 1, 3, 5, and 10 A g^−1^, respectively.

**Figure 11:**
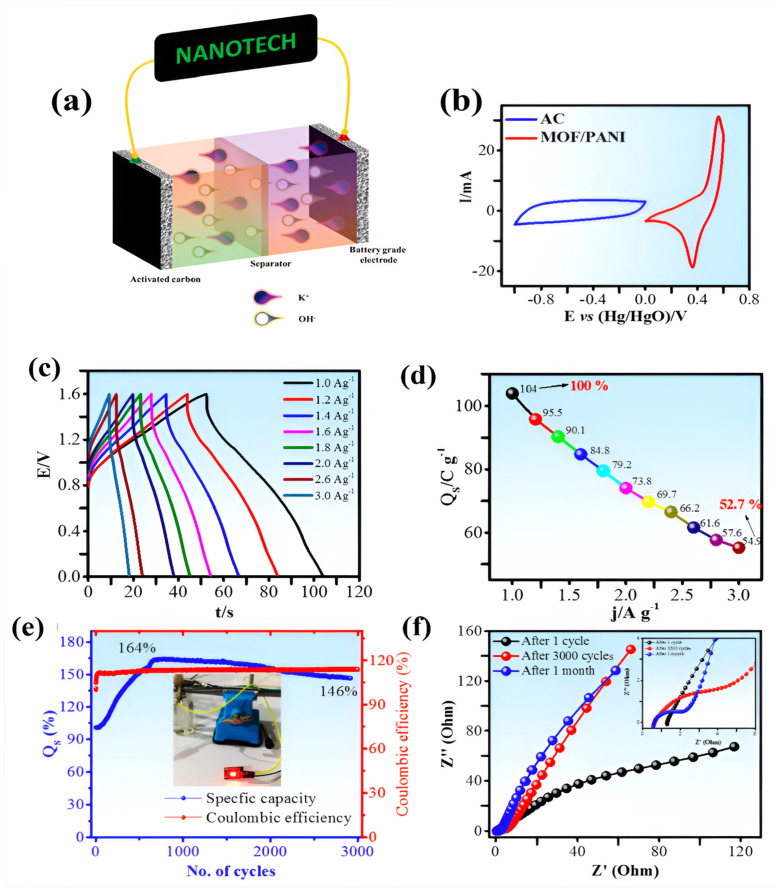
Substrate materials for high-performance supercapattery devices based on co-MOF and polyaniline. (**a**). Pictorial illustration of the ASC device, AC//MOF/PANI; CV profiles of AC anode and MOF/PANI cathode (**b**); GCD curves at various current density (**c**); specific capacity vs. current density plot (**d**); specific capacity and columbic efficiency plot (**e**); and EIS of before stability test, after 3000 GCD cycles and one month later (**f**) [25].

The term “metal organic framework” (MOF) describes a brand new class of porous materials made of robust connections between organic linkers and metal ions. MOFs can exhibit exceptional chemical stability, a huge pore volume, and a large surface area by carefully selecting their constituents. In this instance, we present the cobalt-intercalated MOF/PANI composite for use in supercapattery technology. In cyclic voltammetry at 3 mV s^−1^, MOF/PANI (50/50%) has demonstrated a maximum specific capacity of 154.9 C g^−1^, while at 0.4 A g^−1^, it is 162.5 C g^−1^ shown in Figure 11. Electrochemical impedance spectroscopy revealed that the MOF/PANI (50/50%) combination had the lowest ESR resistance, indicating an increase in conductivity following PANI incorporation. By connecting activated carbon and MOF/PANI, which were separated by a porous membrane, a supercapattery (AC//MOF/PANI) was created. The activated carbon served as the negative electrode while MOF/PANI received positive potential. At a current density of 1.0 A g^−1^, the supercapattery demonstrated a specific capacity of 104.5 C g^−1^. In addition, this hybrid device demonstrated remarkable performance, exhibiting an energy density of 23.2 W h kg^−1^ in accordance with a high power density of 1600 W kg^−1^ at 1.0 A g^−1^. The device can withstand a maximum specific power of 4480 W kg^−1^, and its exceptional stability can be demonstrated by putting it through 3000 GCD cycles at ambient temperature, with a specific capacity of 146%.

**Figure 12:**
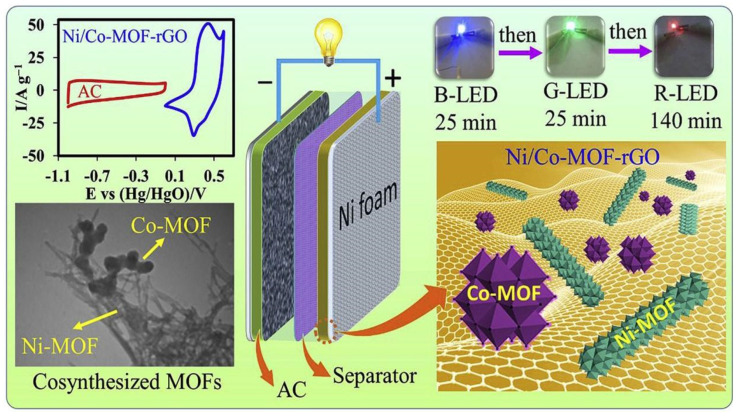
A combination of Ni/Co-MOF-rGO composite as electrode material for high-performance supercapacitors [26].

In a single-step process, a dual Ni/Co-MOF-reduced graphene oxide (rGO) nanocomposite and a novel water-stable Ni-MOF incorporating Co-MOF were synthesized simultaneously within the same reaction vessel. Achieving uniformly distributed MOF particles at the nanoscale level by directly mixing separately synthesized MOFs is challenging. However, introducing both metal precursors and the organic linker into the same reaction vessel ensures homogeneity. The Ni/Co-MOF-rGO nanocomposite exhibits an outstanding specific capacitance of 860 F/g at 1.0 A/g shown in Figure 12 Furthermore, the asymmetric activated carbon/Ni/Co-MOF-rGO device demonstrates an extended cycle lifespan, retaining 91.6% of its capacitance after 6000 charge–discharge cycles at 1 A/g. This device delivers a specific energy of 72.8 Wh/kg at 850 W/kg and retains 15.1 Wh/kg with a high specific power of 42.5 kW/kg.

Because of previously unheard of cumulative CO_2_ emissions, the quantity of carbon dioxide (CO_2_) in the atmosphere is still rising relatively quickly, causing the unfavorable greenhouse gas effect [27]. Undoubtedly, it is becoming increasingly important to create affordable and workable strategies to lower CO_2_ emissions. Hence, the following solutions to this persistent problem are taken into consideration: (i) reducing CO_2_ emissions from stationary and mobile post-combustion sources [28] where CO_2_ concentrations vary from 10% to 15%; and (ii) removing CO_2_ from the air by a process known as direct air capture (DAC), which is an additional option to reduce greenhouse gas emissions globally in a consistent manner. While post-combustion capture is less difficult than DAC, it is acknowledged that DAC may be feasible if an appropriate adsorbent is used that combines the best possible absorption, kinetics, and energetics [28]. Moreover, effective CO_2_ removal at low concentrations is essential for the respiratory systems in cramped areas like aerospace shuttles and submarines to function properly [29].

## 4. Methods of Synthesis for MOF Derivatives

**Figure 13:**
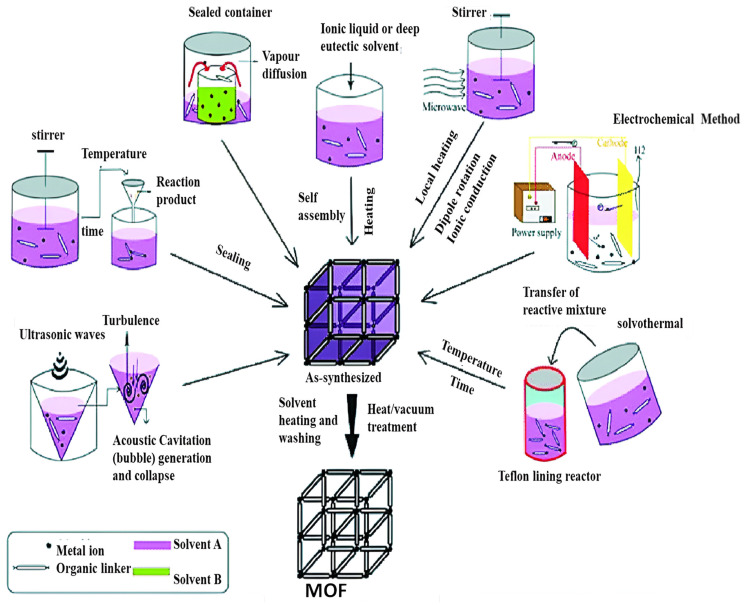
Most commonly employed methods of synthesis of MOFs [30].

The main synthesis techniques of metal–organic frameworks (MOFs) comprise various methods and each method has its own advantages in tailoring the behavior of the resulting material. Some of the most widely employed methods are solvothermal synthesis, hydrothermal synthesis, microwave-assisted synthesis, and mechanochemical synthesis. Some of the most widely used techniques such as solvothermal, hydrothermal, microwave-assisted, and mechanochemical synthesis are discussed in Figure 13.

### 4.1. Solvothermal Synthesis

Solvothermal methods involve chemical reactions in the presence of organic solvents at high temperatures. The reactions are typically carried out in closed vessels like glass vials or acid digestion vessels or stainless steel autoclaves to provide the necessary conditions for successful synthesis [30]. This technique is versatile and enables tuning MOF properties such as crystalline, particle size, and morphology. The main significance of this method lies in the ability to produce well-defined MOF materials suitable for various applications from gas storage to catalysis.

### 4.2. Hydrothermal Synthesis

The hydrothermal method for the synthesis of MOFs is significant for supercapacitor application because of its simplicity and ability to produce MOFs under mild reaction conditions. Generally, in this technique, the precursors are combined in an aqueous solution and subjected to elevated temperatures under pressure. The hydrothermal conditions favor nucleation and growth of MOF crystals with well-defined structures, controlled morphology, and porosity. Hydrothermal synthesis utilizes water as the solvent medium for the reaction. This method relies on the aqueous environment to facilitate the dissolution of metal ions and organic ligands, leading to the formation of MOF crystals.

Many researchers favor hydrothermal methods for synthesizing MOFs, as demonstrated by Gao et al., who employed this technique to produce Ni-MOF vulcanized derivatives. This process yielded nickel sulfides with enhanced electrochemical properties. The findings underscore the efficacy of vulcanizing Ni-MOFs to generate superior electrode materials for high-performance supercapacitors, highlighting the potential of Ni-S2-3 in such applications [31].

### 4.3. Microwave-Assisted Synthesis

Microwave-assisted synthesis has emerged as a promising method for the construction of MOFs tailored for supercapacitor studies. This technique involves microwave irradiation to expedite the synthesis process, resulting in faster reaction kinetics and reduced reaction times compared to other methods. By rapidly heating the reaction mixture, microwave-assisted synthesis enables the precise control of MOF properties such as particle size, morphology, and crystallinity. This control over synthesis parameters allows researchers to optimize the electrochemical performance of MOFs for supercapacitor applications. Moreover, microwave-assisted synthesis offers advantages such as improved yield, enhanced purity, and higher crystallinity of the resulting MOF materials, leading to superior electrochemical performance [31,32].

### 4.4. Mechanochemical Synthesis

Mechanochemical synthesis revolutionizes traditional solution-based methods for crafting MOFs by harnessing mechanical force, often through grinding or milling. This method facilitates bond formation and MOF generation without reliance on solvents or elevated temperatures, achieved by subjecting solid reactants to high-energy impacts. Its advantages include simplicity, scalability, and the ability to tailor highly crystalline MOFs to specific requirements. As it enables efficient and eco-friendly synthesis, mechanochemical synthesis is increasingly favored for MOF production, heralding a promising approach in material creation [33,34,35].

## 5. Constituency Architecture of MOF Nanoarchitecture

The design approach known as constituency architecture applied to MOFs in supercapacitor technology is revolutionizing energy storage. MOFs, renowned for their porous structures and customizable chemical compositions, offer an exciting avenue for boosting supercapacitor performance. Through precise molecular-level design, researchers can customize MOFs’ pore sizes, surface areas, and chemical properties to optimize capacitance, charge/discharge kinetics, and long-term stability. This design strategy provides meticulous control over supercapacitor electrochemical behavior, opening up new possibilities for energy storage efficiency and reliability. By harnessing MOFs’ unique attributes like high surface areas and conductivities, researchers are advancing supercapacitor technology, paving the way for sustainable energy solutions across diverse applications, including portable electronics and electric vehicles. The architecture that is employed in the various dimensions of nanoparticles are discussed in the below sections.

Nanoparticles made of MOFs hold great promise for various applications due to their unique properties. These small, discrete clusters have a carefully controlled size and composition, offering exceptional surface area and the ability to efficiently interact with guest molecules. This nanoarchitecture allows for tailoring functionalities like selective adsorption and catalysis by modifying the ligand chemistry and metal coordination. The confined spaces within the 0D MOF nanoparticles can also enhance guest molecule confinement and diffusion, leading to improved performance in areas like gas storage, sensing, and drug delivery. Furthermore, the small size of these nanoparticles enables their easy integration into composite materials and devices, opening up new avenues for advanced nanotechnology applications. Overall, the nanoarchitecture of 0D MOF nanoparticles provides a versatile platform for developing next-generation materials with enhanced properties and functionalities. Recent work carried out by Wang et al. discussed the advancement in deriving one-dimensional porous and hollow carbon nanofibers from 0D MOFs. The study uses 0D MOFs as precursors for HCNFs (hollow carbon nanofibers) [36]. The study conducted by Qin et al. delves into the magnetic properties of metal–organic frameworks (MOFs) with structures ranging from 0D to 3D based on heptanuclear Co clusters. The compounds exhibit diverse magnetic behaviors, including ferrimagnetic and antiferromagnetic interactions. Compound 1 (0D heptanuclear cluster based on H6L ligand) displays ferromagnetic interactions due to the triangular building blocks constructed by units and formations. In contrast, Compounds 2 (complex that crystallizes in an orthorhombic crystal system with a chiral space group P212121) and 5 (3D microporous structure obtained by introducing isophthalic acid auxiliary ligand) show antiferromagnetic interactions at low temperatures attributed to the anti-anti mode of formates linking clusters. The magnetic properties are influenced by the linking modes of formation between heptanuclear clusters, with Compound 2-derived Co_3_O_4_ showing promising electrochemical performance for applications in batteries [37].

**Figure 14:**
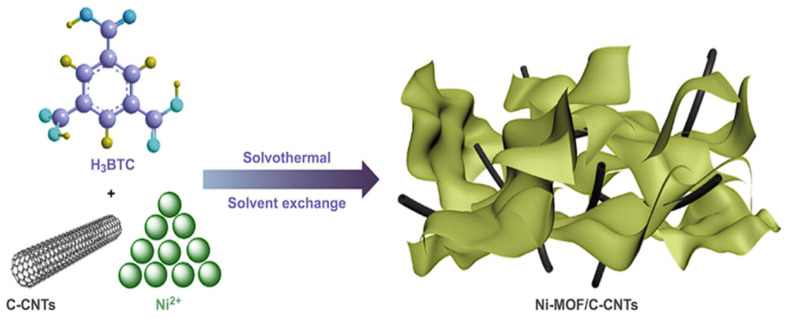
Schematic pathway of 2D Ni-MOF/C-CNTs [38].

Ran et al. concentrated their efforts on fabricating ultrathin nickel metal–organic framework (Ni-MOF) nanosheets infused with functional carboxylated carbon nanotubes (C-CNTs) to improve electrochemical performance. The in situ growth strategy results in the formation of well-interconnected Ni-MOF/C-CNTs hybrid nanosheets shown in Figure 14. Characterization through SEM images reveals the morphological evolution with varying C-CNT content [38].

**Figure 15:**
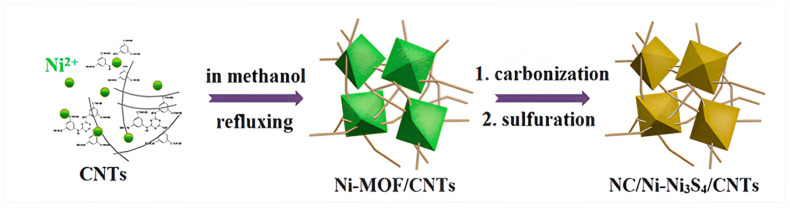
Illustration of synthesis process for composite NC/Ni-Ni_3_S_4_/CNTs.

Yang and his teammates worked on novel MOFs (i.e Ni-MOF) synthesized using a nitrogen-rich ligand and combined with carbon nanotubes (CNTs) to create a Ni-MOF/CNTs composite. This composite served as a precursor for the production of the MOF-derived NC/Ni−Ni_3_S_4_/CNTs composite, where Ni_3_S_4_ nanoparticles were uniformly dispersed within a 3D conductive network shown in Figure 15. The incorporation of rich nitrogen doping and the conductive CNT network enhanced the material’s conductivity, electrolyte penetration, reaction kinetics, coulomb efficiency, and cyclic stability. The study demonstrates the potential of NC/Ni−Ni_3_S_4_/CNTs for high-performance supercapacitors due to its unique structural and electrochemical properties [39].

## 6. Tunable MOF Physical and Chemical Properties

Because of their large pore volumes, relatively high thermal stability, potentially exposed inner surface with high localized charge density, and easily capable programming and modular design (i.e., a given structure with the needed net topology; functionalizable isoreticular structures) from predesigned molecular building blocks (MBBs), metal–organic frameworks (MOFs), a relatively fresh category of porous materials, appear well poised to address the CO_2_ challenge. It is obvious that ongoing research into the separation of novel MBBs will eventually make it easier to rationally create functional MOFs with specific targets. In light of this, our research team keeps searching for new rigid and modular inorganic MBBs. More importantly, though, we want to find reaction conditions that allow us to regularly produce a particular inorganic MBB in situ, a crucial requirement for the future design and logical construction of the desired MOFs.

Using metal–ligand-aimed construction of electron-rich rare-earth (RE) metal ions and noncentrosymmetric heterofunctional ligands with carboxylate and terazolate moieties, we started MOF exploratory syntheses with the goal of creating porous MOFs that had large, restricted charge densities, a potential attribute to promote/enhance the CO_2_ sorption energetics.

**Figure 16:**
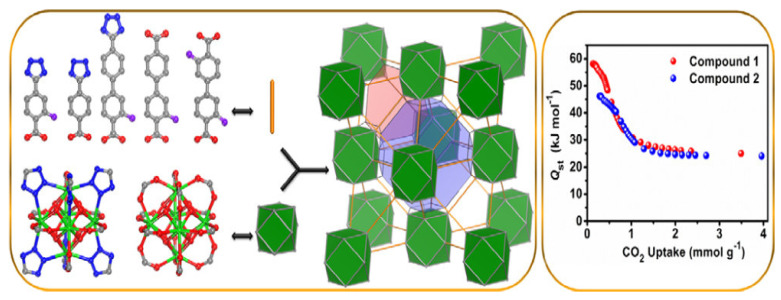
Modifiable rare-earth fcu-MOFs. An integrated framework to boost CO_2_ adsorption energetics and uptake [40].

A series of fcu-MOFs based on linear fluorinated/nonfluorinated, homo/heterofunctional ligands, and rare-earth (RE) metals were targeted and synthesized. The fcu-MOF platform in question was chosen due to its distinct structural features as well as its capacity to control and modify its chemical properties, such as the electron-rich RE metal ion tuning and the high localized charge density that results from the close arrangement of polarizing tetrazolate moieties and fluoro-groups that adorn the exposed inner surfaces of the confined conical cavities. These characteristics allowed for a methodical gas sorption investigation to assess and clarify the impact of different parameters on the energetics of CO_2_–MOF sorption. Our research demonstrates the significance of the combined action of proximal highly concentrated charge density and exposed open metal sites in favor of materials with improved CO_2_ sorption energetic as shown in Figure 16.

### 6.1. Tunable Pore Conditions

MOFs’ inner pore surfaces can be directly functionalized or altered in another way, either during or after assembly. It is possible to create isostructural MOFs by functionalizing the organic linkers or by switching out the metal ions in the inorganic secondary building units (SBUs) for other metal ions. This makes it possible to create MOFs with similar structures but different functions, which can then be used to investigate how the enclosed guest species’ performance is influenced by the chemical environment within the pores. Huang et al. [41] encapsulated Pd NPs in isoreticular (same topology) MOFs with distinctly functioning organic binders (Pd@UiO-66-X, where X = H, NH_2_, or OMe) in order to study the impact of the linker’s electron donation on the embedded Pd NPs’ performance.

### 6.2. Multiple Functional Sites

**Figure 17:**
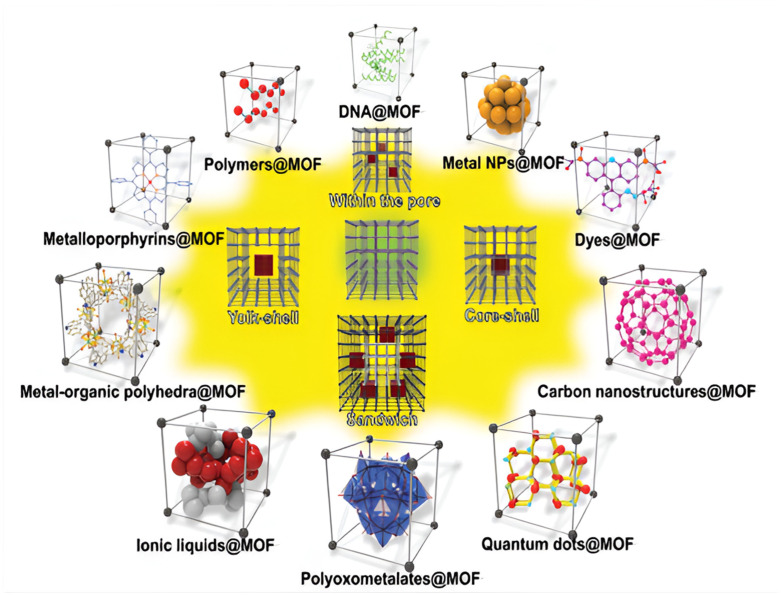
MOFs serve as adaptable carriers for NE encapsulation. NE@MOF composites can be categorized into four groups according to their structural features [43].

By encasing nanoentities (NEs) or altering the linkers and metal nodes, MOF materials can be made functional shown in Figure 17. It is challenging to do in other materials, but the integration of several functional sites into a single system can enable them to perform collaboratively and synergetically. MOFs, for example, are used in optoelectronics, where the metal-containing nodes function as semiconductors and are divided by organic struts that are sensitive to light. By placing active nanoentities into MOFs’ pores, their photocatalytic activity might be improved [42].

Yolk–shell structures have also attracted a lot of attention because of their intricate hierarchical nanostructures. These structures have a unique core@void@shell configuration. Hard-template and template-free techniques have shown to be helpful for designing yolk–shell MOF structures with single-metal cores since the first example was published by Tsung et al. [44]. However, polycrystalline MOFs, which can have flaws or fissures, make up the shells of these nanostructures. The creation of yolk–shell MOF frameworks with just one or multiple metal cores and single crystal MOFs has received a lot of attention lately.

A smart way to achieve good contact between the metal NPs’ whole surfaces and the MOF support is to sandwich them between two layers of MOFs. Zeng et al. [45] reported using this method for the first time. These researchers selected ZIF-8, a typical member of the ZIF subfamily of MOFs, which are based on straightforward zeolite structures, as their host material. Rhombic dodecahedron and cubic hexahedron morphologies of ZIF-8 nanocrystals were coated with Au NPs stabilized with 3-mercaptopropionic acid. The connection between the ZIF-8 phase and the added metal NPs was mediated by the thiol group and carboxylate anion of the organic acid.

According to Minjun Kim et al. [46], the direct carbonization of ZIF-type MOFs, notably ZIF-8, has broadened MOFs’ potential applications, including capacitive deionized saline water production, energy conversion, storage, and electrochemical biosensors. Here, we introduce high-quality ZIF-8 and its modified variants, such as hollow ZIF-8, core–shell ZIF-8@ZIF-67, and ZIF-8@mesostructured polydopamine, in detail.

**Figure 18:**
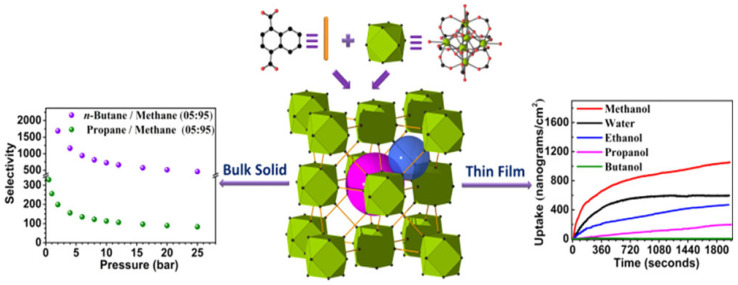
Pore size contraction-based adsorption kinetics-driven gas/vapor separations using modifiable rare-earth fcu-MOF platform [47].

It was possible to intentionally create novel rare-earth fcu MOFs with constrained window apertures by using the selective chemical approach. Achieving the necessary sorbate cutoff through selective and controlled access to the resulting contracted fcu-MOF pores is appropriate for the molecular sieving of gasses and vapors and/or selective adsorption kinetics-based separation shown in Figure 18. As demonstrated in this work for n-butane/methane, butanol/methanol, and butanol/water pair systems, the ensuing RE-1,4-NDC-fcu-MOF structural features, contracted windows/pores, and a substantial concentrations of exposed metal sites, in combination with exceptional hydrothermal treatment and chemical stabilities, yielded notable gas/solvent separation properties, primarily driven by adsorption kinetics.

## 7. Recent Progress on MOF Composites

Some MOF composites and pure MOFs are unstable in diverse pH media and have low conductivity, making them unsuitable for direct use in electrochemical applications. MOFs are regarded as appropriate templates for the post-calcination treatment of metals, compounds made from carbon, and metal composites. However, high temperatures following post-calcination treatment were shown to disrupt the originally ordered structure and cause a loss of intrinsic active sites in MOF. In MOF synthesis, optimizing one parameter appears to jeopardize others. For example, hybridizing MOF and conductive substrates like polyaniline and graphene might prevent MOFs’ inherent micropores from taking part in electrocatalysis. For mechanical assistance and overall improved performance, conductive support is typically used in electrode production.

**Figure 19:**
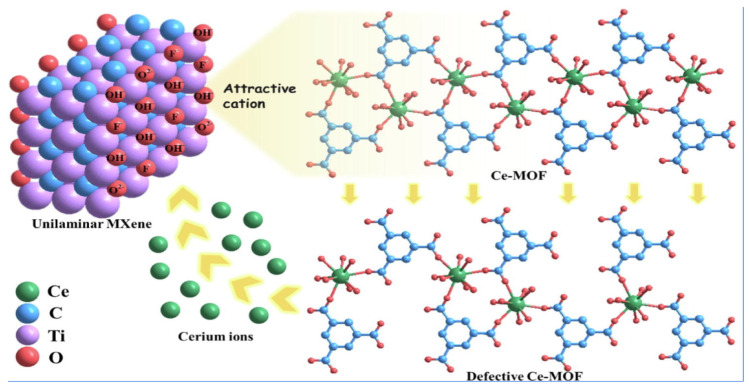
Building MXene @Ce-MOF composites rich in oxygen vacancies for improved energy conversion and storage [48].

By controlling the coordination arrangement and electronic states of atoms, oxygen vacancies can encourage the creation of the surface-active site and raise the electrode material’s conductivity. A structure for MXene@Ce-MOF compounds with lots of oxygen vacancies is presented in this paper shown in Figure 19. Cerium ions are drawn to the hydroxyl groups on the surface of monolayer MXene, resulting in surface defects in Ce-MOF and accelerating the creation of oxygen vacancies. Energy storage capacity and the efficiency of the hydrogen evolution reaction (HER) and oxygen evolution reaction (OER) are both greatly enhanced as a result. The specific capacity of the MXene@Ce-MOF composite is 496 F g^−1^, which is 3.5 times greater than that of MXene alone and 1.8 times greater than that of pure Ce-MOF.

**Figure 20:**
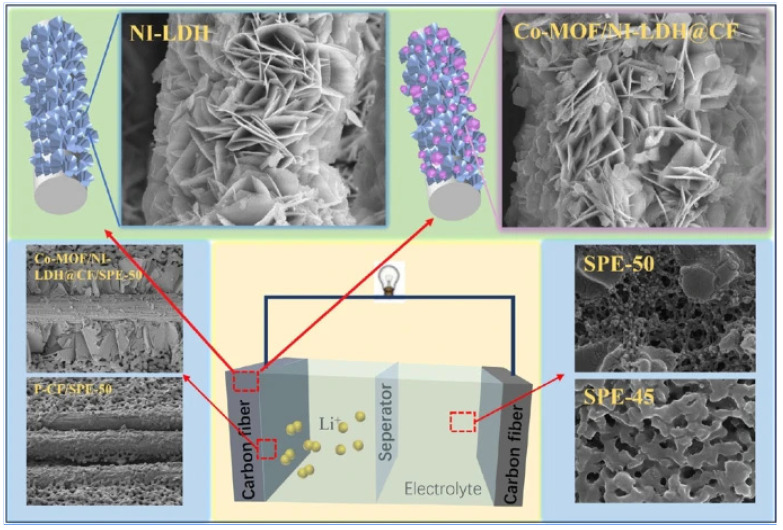
Composites for structural energy storage that are based on improved carbon fiber electrodes with layered double hydroxide metal–organic frame enhancement [49].

Composites with structural energy storage offer the benefit of combining electrochemical and structural strength at the same time. Maintaining appropriate mechanical and electrochemical qualities at an acceptable cost and effort is difficult when using resin structural electrolytes and carbon fiber electrodes in energy storage composites. This article describes a straightforward technique for creating structural supercapacitors using carbon fiber electrodes that were modified to include Ni-layered double hydroxide (Ni-LDH), in situ growth of Co-metal–organic framework (Co-MOF) in two steps (referred to as Co-MOF/Ni-LDH@CF), and a structural electrolyte made of bicontinuous phase epoxy resin shown in Figure 20. In a three-electrode system, the electrode material Co-MOF/Ni-LDH@CF shows better cycle performance (93.3% capacity retention after 1000 cycles) and specific capacity (42.45 F·g^−1^). The structural electrolyte composed of epoxy resin in a bicontinuous phase has an ionic conductivity of 3.27 × 10^−4^ S·cm^−1^. The structural supercapacitor Co-MOF/Ni-LDH@CF/SPE-50 that was fabricated exhibits an energy density of 3.21 Wh·kg−^1^ and a power density of 42.25 W·kg^−1^, all while keeping its tensile strength and modulus at 25.2 GPa and 334.6 MPa, respectively. These findings demonstrate the usefulness of using structural electrolytes made of epoxy resin and modified commercial carbon fiber electrodes for structural energy storage applications.

**Figure 21:**
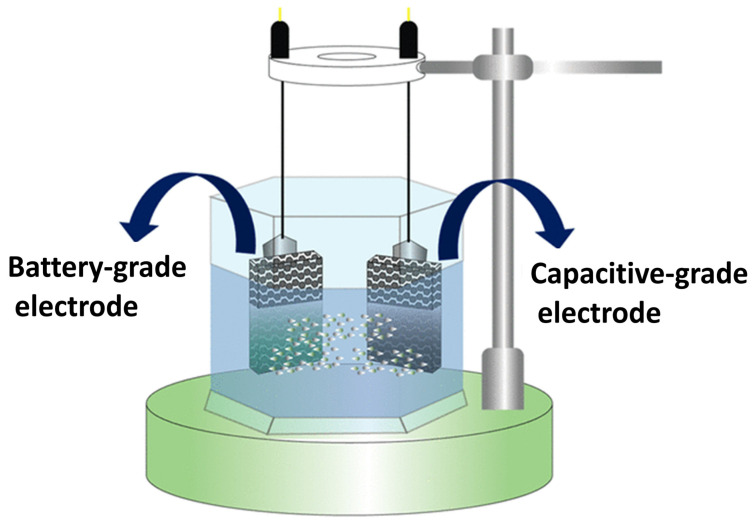
An assessment of both redox-active Cu-MOF and Co-MOF as materials for electrodes for hybrid energy storage devices of battery–supercapacitor type [50].

By combining the excellent power density and cycle stability associated with supercapacitors with the significant amount of energy of batteries, the developing supercapattery provides the best electrochemical performance. A special class of porous substances known as metal–organic frameworks (MOFs) are created by the strong bonding of a metal center with an organic linker. Here, we describe the electrochemical analysis and production of a cobalt- and copper-MOF shown in Figure 21. For the Cu-MOF and Co-MOF, the three-electrode assembly’s initial specific capacities are 451 and 103 C g^–1^ at 3 mV s^–1^, respectively. Cu-MOF/activated carbon is used to create a supercapattery device because of its advantageous properties. The apparatus demonstrates power and energy densities of 2400 W kg^–1^ and 41 Wh g^–1^ accordingly, along with a 99% coulombic efficiency and 92% reversible capacity retention. Additionally, Dunn’s model is modified to clarify the battery–supercapacitor’s diffusive and capacitive contributions. Upcoming energy storage applications may benefit from the Cu-MOF electrode’s advantageous qualities as a battery-grade material.

**Figure 22:**
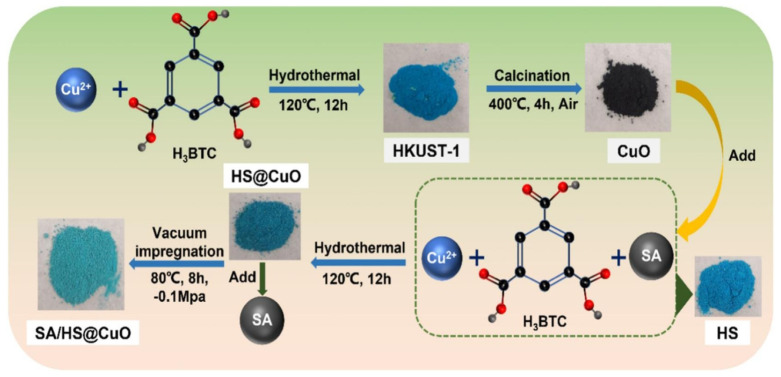
Effective use and storage of solar thermal energy based on phase change substances stabilized by MOF@CuO composites [51].

This work successfully created novel phase change composites based on metal–organic frameworks (MOFs) by a vacuum impregnation process. The SA/HS@CuO phase change composites were made up of the stearic acid (SA) phase change materials and the SA-modified MOF (HS) with CuO produced from MOF (HS@CuO composites) combined to act as support materials shown in Figure 22. The SA leakage across the phase change point could be effectively prevented by HS@CuO composites, which also gave the phase change composites exceptional form stability (SA loading rate: 71.8%) and heat storage capacity (ΔHm: 158.24 J/g). Moreover, the SA/HS@CuO demonstrated exceptional solar thermal conversion capability (η: 95.9%) and significantly enhanced thermal conductivity (3.8 times better than SA). These findings strongly imply that the novel phase change composites have promising futures not only in the domains of thermal storage and management but also in the production and use of solar energy. The supercapacitor performances of various MOF-related materials are reported in Table 1.

## 8. Overview of MOF-Based Materials Market

The global market for metal–organic frameworks (MOFs) is a major participant in many different industries and plays a crucial role in the ecosystem as a whole. Because of their high surface areas and adjustable characteristics, MOFs are porous materials that can be used in a variety of ways for things like medication delivery, gas storage, separation, and catalysis. Their distinct structural properties promote innovation in a variety of fields, including the storage of energy, protecting the environment, and pharmaceuticals. MOFs are essential for improving sustainability and efficiency in the industrial environment. For example, MOFs are used in the energy sector to separate and store gasses, providing a means of producing and storing renewable energy. Furthermore, their uses in catalysis support the development of more environmentally friendly production techniques. MOFs in pharmaceuticals enable regulated drug delivery, transforming the medical industry. Overall, the metal–organic framework market has an impact on more than just a few specific industries; it also has an impact on the industry as a whole by fostering technological and sustainable breakthroughs as well as creative solutions to difficult problems.

The market for metal–organic frameworks has expanded rapidly in the last several years. At a compound annual growth rate (CAGR) of 31.3%, it will increase from THB 0.37 billion in 2023 to THB 0.49 billion in 2024. Strong economic growth in emerging nations, a rise in smartphone usage, scientific advancements in metal–organic frameworks, and a rise in the need for sustainable energy are some of the reasons behind the historical period’s expansion.

There are various reasons why the market for metal–organic frameworks, or MOFs, is expanding significantly. First off, the adoption of MOFs was fueled by the growing need for renewable energy as well as the necessity for effective gas separation and storage. Because of their improved gas adsorption qualities, MOFs are perfect for uses like carbon capture, hydrogen storage, and natural gas storage. The need for MOFs in catalytic applications was also spurred by the growing chemical and pharmaceutical industries. MOFs have the potential to be effective catalysts for a wide range of chemical reactions, leading to higher selectivity and reaction speeds.

The metal–organic framework (MOF) market was significantly impacted by the COVID-19 pandemic. Production delays and disruptions to supply chains resulted from the worldwide lockdowns and limitations implemented to stop the virus’s spread. This caused variations in MOF availability, which hampered the expansion of the market. Furthermore, the economic downturn and uncertainty brought on by the pandemic caused a variation in the demand for MOFs across various businesses. But in the upcoming years, it is anticipated that the demand for MOFs will stabilize and experience significant expansion as economies and industries pick up steam.

**Figure 23:**
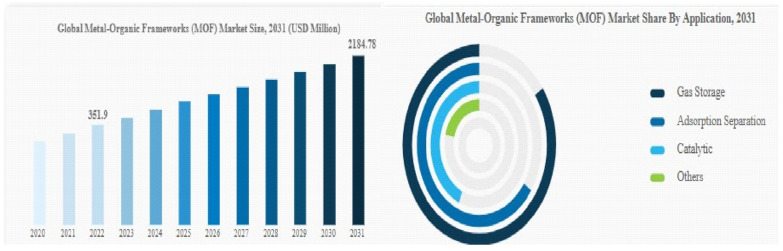
Global MOF for marketing field with various applications.

The market for metal–organic frameworks (MOFs) has bright growth prospects, but there are some obstacles that could prevent it from expanding shown in Figure 23. The technical difficulty in synthesizing MOFs and the scalability are two of the main obstacles. Creating MOFs with the right qualities can be difficult and time-consuming. Furthermore, scaling up MOF production to satisfy market demands continues to be difficult, which restricts the technology’s broad adoption across a range of industries. Furthermore, the MOF market is constrained by cost issues. MOFs may not be as affordable for some applications due to the high costs associated with the process of synthesis and purification steps required in their production. In order to make MOF manufacturing more commercially feasible, efforts are being made to create synthesis techniques that are less expensive and to increase the scalability of MOF production. A comparison of the benefits and drawbacks of MOF methods of synthesis are mentioned in Table 2.

## 9. Conclusions and Future Outlook

In summary, the controlled structures characterized by high surface areas, extensive pore volumes, and adjustable porosities render MOFs and MOF-derived materials highly promising for applications in energy storage and space exploration. The development of MOFs and their derivatives offers encouraging prospects for the synthesis and design of new materials with improved electrochemical properties. But there is still a long way to go in terms of MOF synthesis and different procedures. Consequently, more research on MOFs is needed to create cutting-edge materials for energy conversion and storage and space applications.

Various synthetic techniques, MOFs’ consistency architecture, their tunable chemical and physical properties, and their most recent advancements for energy-related applications are all methodically and thoroughly explained and addressed.

The practical application of MOFs and carbon materials generated from MOFs still faces a number of obstacles. First, more improvement is needed to ensure that MOFs are stable in the air, allowing for their usage in any environment. Moreover, MOFs should be easily and primarily prepared in the air, which would aid in their affordable production and practical market availability. It is essential to enhance the electrical conductivity of MOFs and materials generated from MOFs in order to achieve higher capacity and rate performance in rechargeable batteries and supercapacitors. Enhancing the electrical conductivity of MOFs’ composite electrodes through the incorporation of carbon elements is a fortunate tactic. Even if the many forms and morphologies of carbon materials made from MOFs have revealed their superior electrochemical qualities, the quantity of products they have produced is insufficient. Thus, to achieve high-performance electrode materials for highly wanted energy storage technologies and cost-effective, scalable manufacturing, their production methodologies, remarkable morphology, structure, and reactions chemistry should be explored. In the end, carbon materials produced from MOFs and their composites are playing a growing role in the advancement of supercapacitors and high-performance batteries.

## Figures and Tables

**Table 1:** Literature of MOF based composites and their supercapacitor performances.

MOF Materials	Specific Capacitance (F/g)	Energy Density (Wh/kg)	Power Density W·kg^−1^	Ref.
Ni/Co-MOF@aminated MXene	1924	98	600	[52]
NiCo-MOF@PNTs composite	1109	41.2	375	[53]
NiS/Ni-MOF	787.8	64.67	3200	[54]
MOF-derived nitrogen-doped carbon polyhedron/graphitic carbon nitride	495	11.89	247	[55]
Ni/Co-MOF	758	20.7	800	[56]
NiCo-LDH/Co9S8	800	38	2438	[57]
Ni-MOF	1057	21.05	6030	[58]

**Table 2:** A comparison of the benefits and drawbacks of MOF methods of synthesis.

Synthesis Methods	MOF Materials	Advantages	Disadvantages	Ref.
Microwave-assisted	MOF-74 (Ni)	The process offers reduced crystallization time, improved energy efficiency, ease of controlling reaction conditions, and the ability to control particle size based on precursor concentration.	The process is difficult to implement in the industry, and isolating single crystals is nearly impossible	[59]
Mechanochemical	MOF-74.	The method requires only mechanical forces, avoids extreme operating conditions, and operates without the need for solvents.	Obtaining single crystals for diffraction studies is challenging, and the product often contains secondary phases.	[60]
Hydrothermal/solvothermal	Hf-MOFs	The process facilitates easy technology transfer to the industry, allows precise control of crystal growth, and operates effectively across a wide temperature range.	Expensive running costs and a lengthy synthesis period.	[61]
Sonochemical/ultrasonic	Cu-BTC	The method can achieve homogeneous crystal size and morphology and can be used to isolate a pure phase.	Large single crystals that are required for diffraction studies.	[62]
Slow diffusion	IRMOF-1	The process enables the preparation of large single crystals and requires ambient or low temperatures.	Synthesis could take a few days. The amount of the product lessens.	[63]
Electrochemical	Zn-MOF	The method offers ease of industrial application, a short synthesis time, and uses current and voltage to control morphology.	The structure and morphology need exact control since they are extremely sensitive to electrolyte, voltage, and current conditions.	[64]
In situ synthesis	(Zn_4_O(BDC)_3_	Simple to perform; just mild reaction conditions are needed.	The requirements for simultaneous synthesis and integration make deployment on an industrial scale challenging.	[65]
